# When Small Cells Matter: Diagnosing Bladder Small Cell Carcinoma on Urine Cytology

**DOI:** 10.1111/cyt.70081

**Published:** 2026-04-20

**Authors:** Haley Corbin, Samer Khader, Gabriela Quiroga‐Garza, Liron Pantanowitz

**Affiliations:** ^1^ Department of Pathology UPMC/University of Pittsburgh School of Medicine Pittsburgh Pennsylvania USA

**Keywords:** bladder, cytology, neuroendocrine, small cell carcinoma, urine

## Abstract

Small cell carcinoma of the urinary bladder (SCCB) is a rare and highly aggressive neuroendocrine neoplasm that poses significant diagnostic challenges, particularly on urine cytology. We report a case of a 68‐year‐old man with painless hematuria in whom SCCB was initially detected on voided urine cytology. Cytologic evaluation revealed small malignant cells with high nuclear‐to‐cytoplasmic ratios, nuclear moulding, finely granular chromatin, necrotic background and apoptotic debris, raising suspicion for a neuroendocrine carcinoma. Immunohistochemical studies performed on the urine cell block supported this diagnosis. Subsequent transurethral resection demonstrated a predominant high‐grade neuroendocrine carcinoma admixed with conventional high‐grade urothelial carcinoma, confirming primary bladder origin. This case highlights the importance of careful cytomorphologic assessment, the diagnostic value of cell block immunohistochemistry and the limitations of the Paris System in classifying non‐urothelial malignancies encountered in urine specimens.

## Graphical abstract

Small cell carcinoma of the bladder is rare, aggressive and diagnostically challenging on urine cytology. We report a case detected in urine and confirmed by cell block immunohistochemistry, highlighting the importance of careful assessment and limitations of the Paris System.

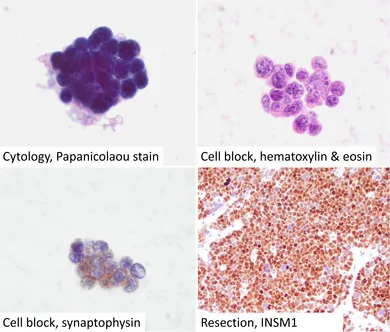

## 1 Introduction

Neuroendocrine neoplasms occur extremely rarely in the bladder. Small cell carcinoma of the urinary bladder (SCCB) is an aggressive neuroendocrine carcinoma that represents less than 1% of all bladder malignancies with a median survival of 11 months. It predominantly affects elderly men with a history of smoking and often presents with painless hematuria [1, 2].

SCCB diagnosis is challenging given its rarity in urine cytology and frequent crush artefact on biopsy specimens. Further, gross, clinical and radiologic presentation can be identical to urothelial carcinoma, and these tumours often coexist [2, 3].

We report a case of SCCB initially detected by urine cytology, highlighting the importance of careful cytomorphologic evaluation, the utility of cell block preparation and the limitations of the Paris system for reporting urine cytology in non‐urothelial tumour classification.

## 2 Case Presentation

A 68‐year‐old man with a 50 pack‐year smoking history complained of a one‐month history of intermittent, painless hematuria associated with urinary urgency and frequency.

A contrasted CT scan of the abdomen and pelvis showed a left posterolateral intravesical mural polypoidal mass (Figure 1). No extravesical extension or pathologically enlarged pelvic or abdominal lymph nodes were seen.

**FIGURE 1:**
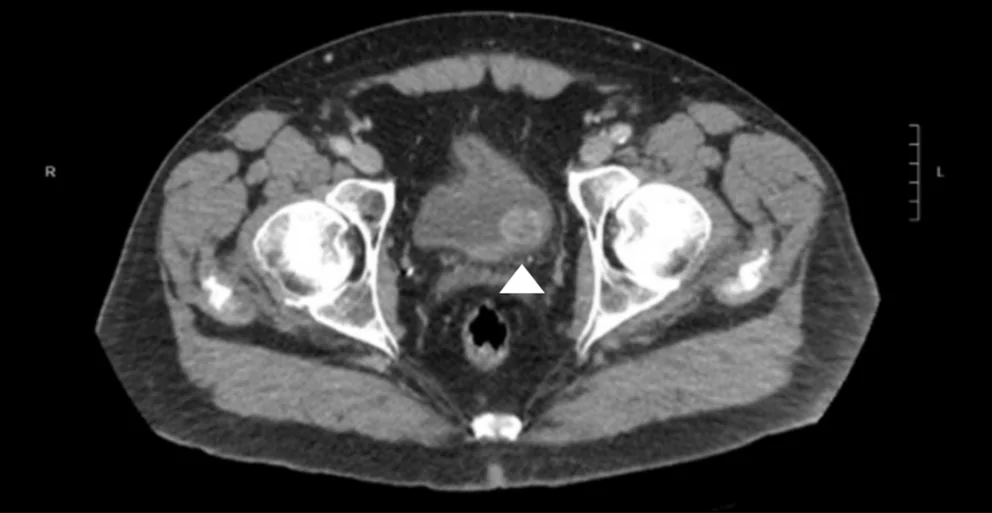
CT abdomen and pelvis with contrast showing a left posterolateral intravesical mural mass (arrowhead).

Cystoscopy corroborated CT findings. Voided urine was collected (70 cc grossly non‐bloody urine in 10 cc CytoLyt), and a ThinPrep Papanicolaou stained slide was prepared which was hypocellular with a bloody and necrotic background. The urine contained a few clusters of small, highly atypical and degenerated epithelial cells. The nuclei of these neoplastic cells were round to oval with irregular nuclear membranes, finely granular chromatin and inconspicuous nucleoli with scant cytoplasm (Figure 2). Nuclear moulding, necrotic debris, and scattered apoptotic bodies were present. The presence of a second population of neoplastic cells characteristic of high‐grade urothelial carcinoma was not appreciated. Immunohistochemical (IHC) testing was performed on a formalin‐fixed cell block (results in Table 1). The overall findings favoured high‐grade carcinoma with neuroendocrine features.

**FIGURE 2:**
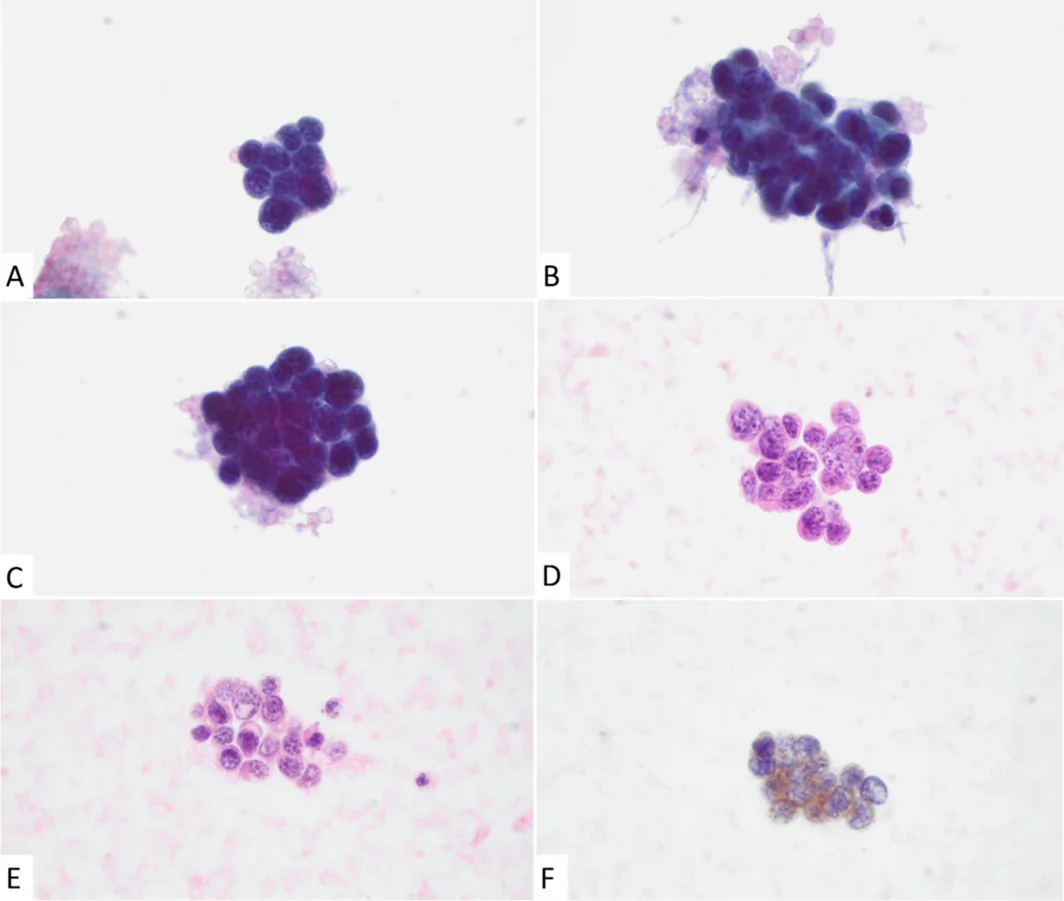
ThinPrep Pap‐stained cytologic preparation of urine containing clusters of small cells with high nuclear‐to‐cytoplasmic ratios, hyperchromasia, finely granular chromatin, nuclear moulding, and scant cytoplasm with background necrosis (A–C, Papanicolaou stain, 60× magnification). The cell block preparation shows loose clusters of these epithelioid cells with nuclear pleomorphism and granular chromatin (D, E, H&E, 60× magnification). The tumour cells are positive for synaptophysin (F), supporting neuroendocrine differentiation (immunohistochemistry, 60× magnification).

**TABLE 1:** Immunohistochemical profile of neuroendocrine carcinoma cells in urine cytology and in the neuroendocrine and urothelial carcinoma components seen in the resection specimen.

	Immunohistochemical stain	Stain result
Urine cell block sample	Pankeratin	Positive
	Synaptophysin	Positive
	GATA3	Negative
	LCA	Negative
	Androgen receptor	Negative
	NKX3.1	Negative
	CD56	Negative
	Chromogranin A	Negative
	INSM1	Negative
	POU2F3	Negative
	TTF1	Negative
	Vimentin	Negative
	Ki67	Slightly increased proliferation index (10%–20%)
Resection sample		
Neuroendocrine carcinoma component	CAM5.2	Positive (paranuclear, dot‐like)
	CK7	Positive (paranuclear, dot‐like)
	Synaptophysin	Positive (diffuse)
	INSM1	Positive (diffuse)
	AR	Positive (weak)
	P63	Positive (weak)
	Ki67	High proliferation index (95%)
	CK20	Negative
	NKX3.1	Negative
	PSA	Negative
	GATA3	Negative
	Chromogranin	Negative
Urothelial carcinoma component	CAM5.1	Positive
	CK7	Positive
	CK20	Positive
	P63	Positive
	GATA3	Positive
	AR	Positive
	Chromogranin	Negative
	Synaptophysin	Negative
	INSM1	Negative
	NKX3.1	Negative
	PSA	Negative

A subsequent transurethral resection demonstrated two distinct tumour components: a predominant (90%) high‐grade neuroendocrine carcinoma and a conventional high‐grade papillary urothelial carcinoma. The presence of urothelial carcinoma in situ supported primary urothelial origin of this tumour. The neuroendocrine component showed relatively small cells with high nuclear‐to‐cytoplasmic ratios, nuclear moulding, salt and pepper chromatin, and scant amphophilic cytoplasm, arranged in solid nests, cords and trabeculae (Figure 3). High mitotic activity (> 20 per high‐power field), frequent apoptosis, areas with crush artefact and widespread geographic necrosis were present. IHC results on the resection specimen are listed in Table 1.

**FIGURE 3:**
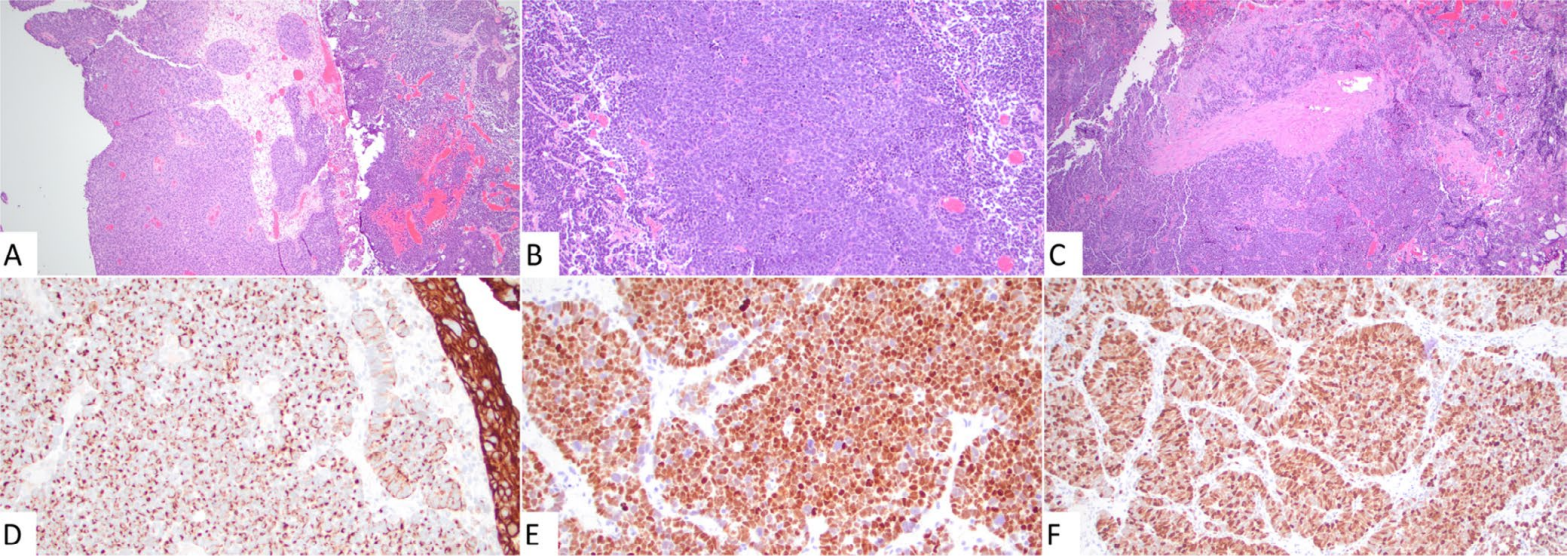
Transurethral resection of the bladder tumour showing a component of urothelial carcinoma (A, left, H&E, 4× magnification) with adjacent fragments showing clusters and sheets of carcinoma with high nuclear‐to‐cytoplasmic ratios and finely granular cytoplasm, consistent with small cell carcinoma (A, right, H&E, 4× magnification & B, H&E, 10× magnification) invading extensively into detrusor muscle (C, H&E, 4× magnification). The neuroendocrine component shows paranuclear, dot‐like staining for CK7 with overlying urothelial carcinoma in situ showing strong membranous CK7 staining (D, immunohistochemistry, 20×). The neuroendocrine component shows INSM1 nuclear staining (E, immunohistochemistry, 20×) with high Ki67 proliferation index (F, immunohistochemistry, 10×).

Oncomine next‐generation sequencing demonstrated an RB1 mutation (p.S149*), TERT promoter mutation (C228T), PT53 mutations, RB1 copy number loss and 19q region copy number loss (partial deletion). The tumour was microsatellite stable with high tumour mutational burden, supporting the diagnosis of small cell carcinoma.

Neoadjuvant chemotherapy and immunotherapy were initiated, with plans for radical cystoprostatectomy.

## 3 Discussion

Our case highlights key diagnostic features of SCCB on urine cytology. The cytomorphology correlated with the predominant high‐grade neuroendocrine carcinoma morphology seen on resection. The presence of several small clusters of neoplastic cells with neuroendocrine features and background necrosis was highly concerning in the urine cytology sample. Although rosettes and nuclear streaking, often helpful for diagnosing small cell carcinoma, are usually absent in urine samples, we did observe substantial crushed cells in the resection, which might be seen on cytology as single cells stretched between clusters [4]. Tumour cells in the urine were positive for pankeratin and synaptophysin while negative for INSM1, which was diffusely positive in the tumour in the resection specimen, potentially related to alcohol‐fixation and degenerative change of the urine sample.

Urine cytomorphology of SCCB is akin to that of small cell carcinoma at other body sites, showing the above described cytologic features [5, 6, 7, 8, 9, 10, 11]. Background necroinflammatory debris and naked nuclei have been reported [5, 8, 11, 12, 13]. The presence of a second population of cells with large vesicular nuclei and conspicuous nucleoli, as well as cell‐in‐cell tight nuclear moulding with the appearance of triplet signet ring cells may be two additional helpful features [4]. The presence of cell clustering, nuclear moulding and hyperchromasia together is most useful to distinguish small cell carcinoma from the top differential diagnoses of small cell neoplasms in urine cytology, namely, lymphoproliferative disorders and melanoma [14].

In cases of non‐urothelial neoplasms, or when urothelial cells are not well represented, the Paris system may be suboptimal, and the use of general cytology diagnostic categories may be more appropriate. In the setting of a bladder mass and cytology showing malignant cells with neuroendocrine features, distinguishing between a pure neuroendocrine carcinoma, a mixed high‐grade urothelial carcinoma or an extravesical primary neoplasm may not be possible (Table 2). As the Paris system does not include a category of suspicious for a non‐urothelial malignancy [15], this case was classified as suspicious for malignancy, using general cytology terminology with an explanatory comment rather than assigned a Paris system category.

**TABLE 2:** Small cell carcinoma versus high‐grade urothelial carcinoma in urine cytology.

Feature	Small cell carcinoma	High‐grade urothelial carcinoma
Typical clinical context	Rare; often associated with aggressive bladder tumour; may present with paraneoplastic syndromes	Most common malignant diagnosis in urine cytology
Cellularity	Often high, but cells may be fragile	Usually high, with abundant malignant cells
Architecture	Single cells, loose clusters; nuclear moulding common	Discohesive cells ± clusters, sheets
Cell size	Small, ~lymphocyte‐sized	Larger, pleomorphic
N:C ratio	Very high	High, but less extreme
Nuclear shape	Round to oval, moulded, overlapping	Irregular, angulated, pleomorphic
Chromatin	Finely granular (‘salt‐and‐pepper’)	Coarse, clumped, hyperchromatic
Nucleoli	Absent or inconspicuous	Often present, may be prominent
Cytoplasm	Scant, delicate	Moderate, often dense
Background	Necrosis, apoptotic debris, crush artefact	Necrosis common; tumour diathesis
Mitoses/apoptosis	Frequent	Frequent
Nuclear moulding	Common and characteristic	Uncommon
Crush artefact	Common	Uncommon
Diagnostic pitfall	Can mimic lymphoma or degenerated cells	Can overlap with CIS, treatment effect
Helpful IHC (cell block)	Synaptophysin+, Chromogranin+, INSM1+, CD56+, Ki‐67 high	GATA3+, p40/p63+, CK7/CK20+
Clinical significance	Highly aggressive, systemic therapy used	Aggressive but typically surgically managed

Given the rarity of SCCB, metastasis from another site, such as lung, should be considered when identified in a urine specimen. Immunophenotype of a metastasis can be indistinguishable from SCCB (e.g., TTF‐1 positivity is seen in some SCCBs) requiring clinical and radiologic correlation for primary site identification [16]. Cases of small cell carcinoma of the lung, cervix, prostate and other sites have been diagnosed by urine cytology [4, 17]. as well as a rare case of ovarian small cell carcinoma, with the absence of a neoplastic urothelial component and positive immunohistochemical staining for WT1 supporting ovarian primary site [16]. Thus, immunohistochemical testing is an integral part of the workup of neuroendocrine carcinomas identified in urine cytology. However, clinical correlation may ultimately be needed for primary site identification, and the presence of an associated urothelial component can be helpful to suggest bladder primary.

## 4 Conclusions

We report a case of SCCB diagnosed on urine cytology, highlighting the classic cytomorphologic features of a rare entity infrequently encountered in urine specimens. Recognition in urine specimens is crucial as SCCB closely mimics urothelial carcinoma clinically and radiologically, and both might first be detected in a urine sample. However, SCCB carries a dramatically different prognosis and management, representing more aggressive disease which often presents at advanced stage requiring systemic therapy [3, 4, 5, 9, 12, 18].

## Author Contributions

**Haley Corbin:** conceptualization, writing – original draft. **Samer Khader:** conceptualization, supervision, writing – review and editing. **Gabriela Quiroga‐Garza:** conceptualization, supervision, writing – review and editing. **Liron Pantanowitz:** conceptualization, supervision, writing – review and editing.

## Funding

The authors have nothing to report.

## Conflicts of Interest

The authors declare no conflicts of interest.

## Data Availability

Data sharing is not applicable to this article as no datasets were generated or analysed.
